# Brain-derived neurotrophic factor in fibromyalgia: A systematic review and meta-analysis of its role as a potential biomarker

**DOI:** 10.1371/journal.pone.0296103

**Published:** 2023-12-21

**Authors:** Amir Hossein Behnoush, Amirmohammad Khalaji, Shaghayegh Khanmohammadi, Parsa Alehossein, Behrad Saeedian, Parnian Shobeiri, Antonio L. Teixeira, Nima Rezaei

**Affiliations:** 1 School of Medicine, Tehran University of Medical Sciences (TUMS), Children’s Medical Center Hospital, Dr. Qarib St., Keshavarz Blvd, Tehran, Iran; 2 Network of Immunity in Infection, Malignancy and Autoimmunity (NIIMA), Universal Scientific Education and Research Network (USERN), Tehran, Iran; 3 Non–Communicable Diseases Research Center, Endocrinology and Metabolism Population Sciences Institute, Tehran University of Medical Sciences, Tehran, Iran; 4 Research Center for Immunodeficiencies, Pediatrics Center of Excellence, Children’s Medical Center, Tehran University of Medical Sciences, Tehran, Iran; 5 Department of Radiology, Memorial Sloan Kettering Cancer Center, New York, NY, United States of America; 6 Neuropsychiatry Program, Department of Psychiatry and Behavioral Sciences, McGovern Medical School, The University of Texas Health Science Center at Houston, Houston, TX, United States of America; 7 Department of Immunology, School of Medicine, Tehran University of Medical Sciences, Tehran, Iran; Chiba Daigaku, JAPAN

## Abstract

**Background:**

Fibromyalgia (FM) is a form of chronic pain disorder accompanied by several tender points, fatigue, sleeping and mood disturbances, cognitive dysfunction, and memory problems. Brain-derived neurotrophic factor (BDNF) is also a mediator of neurotrophin for many activity-dependent processes in the brain. Despite numerous research studies investigating BDNF in FM, contradictory results have been reported. Thus, we investigated the overall effect shown by studies to find the association between peripheral BDNF concentrations and its gene polymorphisms with FM.

**Methods:**

A systematic search in online international databases, including PubMed, Cochrane Library, Embase, the Web of Science, and Scopus was performed. Relevant studies assessing BDNF levels or gene polymorphism in patients with FM and comparing them with controls were included. Case reports, reviews, and non-English studies were excluded. We conducted the random-effect meta-analysis to estimate the pooled standardized mean difference (SMD) or odds ratio (OR) and 95% confidence interval (CI).

**Results:**

Twenty studies were found to be included composed of 1,206 FM patients and 1,027 controls. The meta-analysis of 15 studies indicated that the circulating BDNF levels were significantly higher in FM (SMD 0.72, 95% CI 0.12 to 1.31; p-value = 0.02). However, no difference between the rate of Val/Met carrier status at the rs6265 site was found (p-value = 0.43). Using meta-regression, the sample size and age variables accounted for 4.69% and 6.90% of the observed heterogeneity of BDNF level analysis, respectively.

**Conclusion:**

Our meta-analysis demonstrated that FM is correlated with increased peripheral BDNF levels. This biomarker’s diagnostic and prognostic value should be further investigated in future studies.

## 1. Introduction

Fibromyalgia (FM) is a chronic, centralized pain syndrome characterized by disordered processing of painful stimuli with a higher frequency among women [1]. It is accompanied by several tender points, fatigue, sleeping and mood disturbances, memory problems, and cognitive dysfunction. FM is the second most common rheumatic disorder after osteoarthritis and affects 2–8% of the general population, with a greater frequency among women [2–4]. Despite the ongoing efforts to develop more objective criteria for diagnosing FM [4–6], criteria are still subjective, and symptoms are nonspecific and overlap with other rheumatological diseases [7, 8]. Currently, there is no reliable laboratory marker to diagnose FM [5, 9].

Although the definite pathophysiology of FM remains elusive, FM often coexists with overlapping conditions termed "central sensitivity syndromes", such as chronic fatigue syndrome. It is thought to rely on altered neuroplasticity that induces pain amplification. This might be raised by increased excitability of central nervous system mechanisms regardless of peripheral inflammation or neural lesion, leading to pain hypersensitivity [10–14]. Therefore, it has been postulated a possible role for neuroplasticity mediators in the pathophysiology of FM.

Brain-derived neurotrophic factor (BDNF), as a member of the neurotrophin family, mediates the regulation of synaptic plasticity, promoting the growth, survival, and development of neurons [15–18]. BDNF has been suggested in the transmission and modulation of pain [19], facilitating glutamatergic excitability while inhibiting gamma-aminobutyric acid (GABA)ergic responses [20]. Higher levels of BDNF can induce hyperalgesia via N-methyl-D-aspartate (NMDA) receptor-dependent pathways [21, 22] and are related to a lower pressure-pain threshold in FM [23].

BDNF shows sensitizing capacity in numerous levels of the pain pathways, such as spinal cord neurons, peripheral nociceptors, and the brain [16]. BDNF gene is also located on chromosome 11p14.1 with epigenetic control for its expression [24]. Single nucleotide polymorphism is the most often studied gene polymorphism. The G to A transition at position 196 in exon 5 of rs6265 (BDNF gene) leads to a substitution of valine (Val) with methionine (Met) at BDNF peptide sequence precursor codon 66, commonly referred to as Val66Met [25]. Subsequently, the Met allele can impair the extracellular BDNF levels regulation [26].

Several studies have investigated circulatory BDNF among FM patients compared to healthy individuals. There are discrepancies in the results found by the studies since some reported higher levels of BDNF in patients with FM, some reported lower levels, and some did not find any significant difference. A previous systematic review of seven studies showed that peripheral circulatory BDNF levels are higher in patients with FM [8]. Nevertheless, more recent studies reported either no significant difference or lower BDNF when comparing FM and healthy control groups [27–29]; moreover, the association between BDNF gene polymorphism and FM was not assessed. Hence, the profile of BDNF associated with FM remains to be determined. Building upon the existing knowledge gaps and controversies surrounding BDNF in FM, the primary aim of our current study is to systematically investigate the potential correlation between peripheral BDNF levels and its gene polymorphisms with FM. We wanted to examine whether BDNF is associated with FM or has any role in diagnosis, prognosis, and quality of life in patients with FM.

## 2. Methods

### 2.1. Search strategy

Our systematic review was performed with adherence to Preferred Reporting Items for Systematic Reviews and Meta-Analyses (PRISMA) [30] guidelines (S1 Table). A systematic comprehensive search in international online databases, including PubMed, Embase, the Web of Science, Scopus, and Cochrane Library was performed in July 2023. Keywords used were “brain-derived neurotrophic factor” AND “fibromyalgia” and other relevant keywords, which were described in detail in S2 Table. No filters or limitations were applied to the search. The protocol of our current review was registered at PROSPERO (CRD42022361602).

### 2.2. Inclusion and exclusion criteria

The inclusion criteria for our review were: 1) studies reporting BDNF serum, plasma, or cerebrospinal fluid (CSF) levels in FM and controls and 2) studies investigating BDNF rs6265 gene polymorphism, as the most studied gene polymorphism, in patients with FM and controls. Also, we excluded: 1) studies not having a control group without FM; 2) studies without reporting BDNF levels (the corresponding authors were contacted, and if no data could be obtained the study was excluded); 4) case reports, conference abstracts, and reviews; and 3) non-English investigations.

### 2.3. Screening

After removing duplicates from the initial search, two reviewers (AHB and AK) independently evaluated titles/abstracts for relevant studies. Then, full texts of studies were assessed for included studies, and in case of any controversy, a discussion with the third reviewer (PS) determined the decision. Finally, references to the included studies were screened.

### 2.4. Data extraction

Using a pre-defined sheet, two authors (AHB and AK) extracted the studies’ data independently, including 1) basic study details (First author’s name, publication location, and year); 2) the baseline demographic features of the studied population (mean age, sample size, and gender distribution in FM and control groups); 3) serum and/or plasma BDNF levels in each study group; 4) BDNF levels in CSF in each group; 5) BDNF gene (rs6265) polymorphism alleles in each group. In cases where data on BDNF levels were not explicit, we contacted the corresponding authors.

### 2.5. Quality assessment

The “Newcastle-Ottawa Quality Assessment Scale” (NOS) for observational studies was used for the investigation of the qualities of the included studies [31]. Two independent authors (AHB and AK) performed the quality assessment. In case of any disagreement, an investigation by the third reviewer (PS) resolved it. The NOS includes three main categories of bias including selection, comparability, and outcome. Scores of 9–10, 7–8, 5–6, and less than 5 were considered “very good”, “good”, “satisfactory”, and “unsatisfactory”, respectively.

### 2.6. Statistical analysis

Random-effect meta-analysis of BDNF concentrations in FM and control groups was conducted to estimate the standardized mean difference (SMD) and 95% confidence interval (CI). For the gene polymorphism meta-analysis, the odds ratio (OR) and 95% CI were used to compare the Met/Val genes in each group. The p-values <0.05 were chosen as the statistically significant cutoff. The quality of evidence and strength of recommendations were assessed based on the GRADE approach that incorporates five domains: risk of bias, inconsistency, indirectness, imprecision, and publication bias [32].

Wherever median and range or median and interquartile range were reported in the studies, we converted them to mean and standard deviations (SDs) using the methods suggested by Luo et al. and Wan et al. [33, 34]. Moreover, combining means and SDs was done when needed based on the Cochrane Handbook suggestion [35].

Higgins’ I-square test based on Cochrane’s Q was used for the calculation of heterogeneity. Heterogeneity of ≤25%, 26–75%, and ≥75% were used as thresholds for low, moderate, and high heterogeneity, respectively. The high heterogeneity among studies led to the use of random-effect meta-analysis (DerSimonian and Laird) [36]. Sensitivity analysis was done by omitting each study (leave-one-out) and investigating the effect on total effect size. Identification of outliers via Galbraith plots was also performed to identify possible outliers. Additionally, meta-regression based on the sample size, publication year, and mean age was also conducted to identify the source of variance among the studies analyzed. Finally, visual inspection of funnel plots for possible asymmetry in addition to statistical tests of Begg’s and Egger’s were used for the assessment of publication bias [37, 38].

## 3. Results

### 3.1. Study characteristics

**Fig 1:**
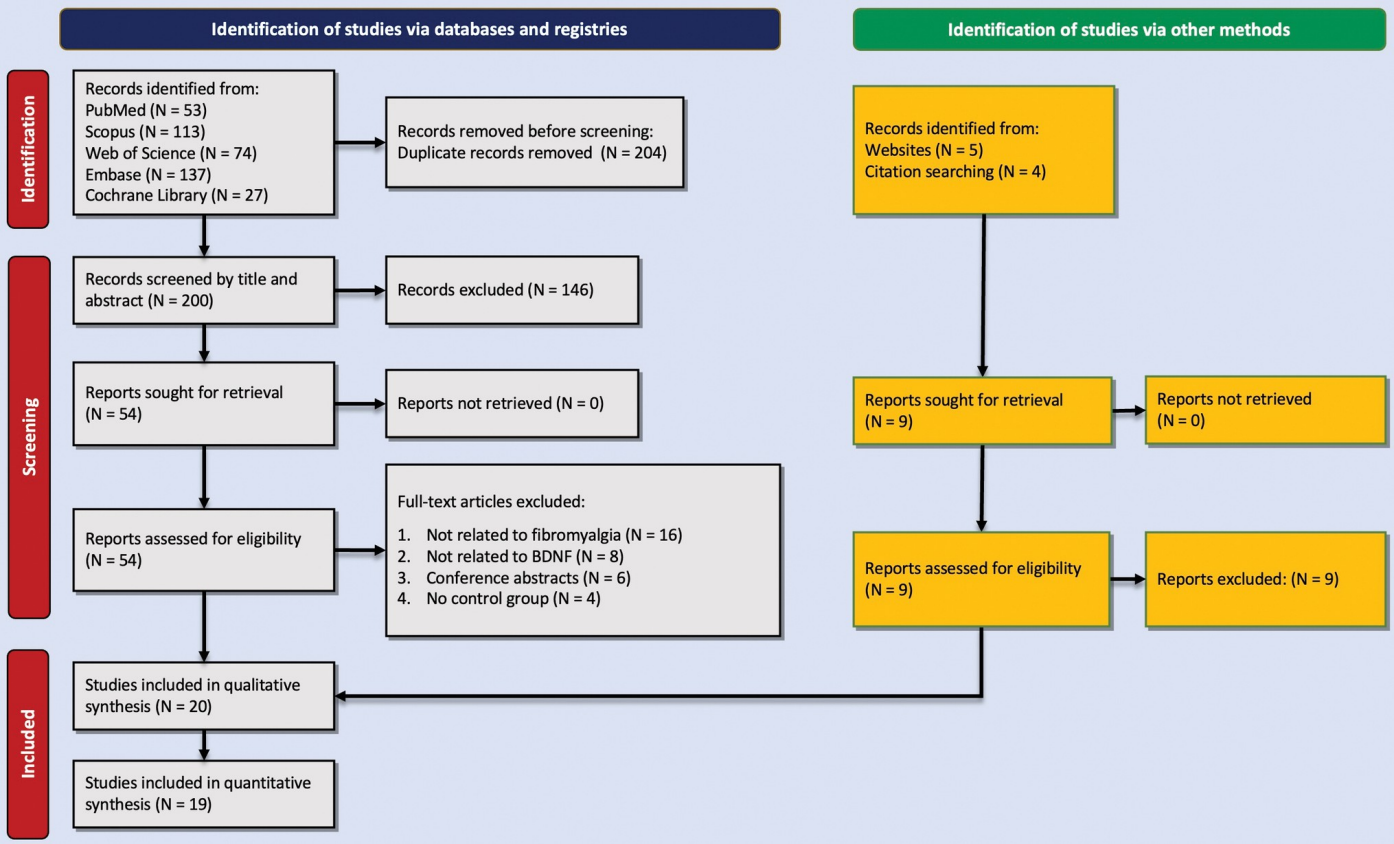
Flow PRISMA diagram representing the selection process of eligible studies.

Our search resulted in 404 records from PubMed (n = 53), Web of Science (n = 74), SCOPUS (n = 113), Embase (n = 137), and Cochrane Library (n = 27). After removing duplicate studies and excluding reports not eligible for this study, 20 studies were included in this review composed of 1,206 patients with FM and 1,027 controls [28, 29, 39–56]. A PRISMA flow chart is available in Fig 1. Nine studies were conducted in Brazil [29, 40–44, 53, 54, 56], two in Italy [28, 49], two in Germany [47, 52], and one study in each of the following countries: Iran [39], Egypt [51], Sweden [55], Korea [45], Belgium [46], Turkey [50], and the United States [48]. Thirteen studies only included females [29, 39–44, 46, 52–56]. Studies evaluated BDNF gene polymorphism [44–46, 48] and/or BDNF concentration in the serum [28, 29, 39–43, 46, 47, 50–53], plasma [54–56], or CSF [49]. American College of Rheumatology (ACR) guidelines of 1990 [57], 2006 [58], 2010 [59], and 2016 [60] were used in six [45, 47, 49, 52, 54, 55], one [56], seven [29, 41–43, 46, 51, 53], and five studies [28, 29, 39, 40, 44], respectively. Details of these included studies in our study are presented in Table 1. All studies were “good” or “very good” given their NOS quality assessment scores. Table 2 provides the details of the quality assessment based on NOS scores.

**Table 1:** Characteristics of studies evaluating the relation between BDNF levels/gene polymorphism and FM.

Author	Year	Design	Location	FM	Control	N total	N FM	Age	BMI	% Female	Main Findings	Source
Alves et al.	2020	Cross-sectional	Brazil	Previous diagnosis of FM in women older than 18 according to 2016 ACR criteria	No chronic pain, FM, systemic lupus erythematosus, arthrosis, rheumatoid arthritis, or use of antidepressant drugs	216	108	49.8 ± 9.5	27.6 ± 4.8	100	The frequency of the Val allele was significantly higher in patients with FM (90.3%) compared to controls (83.8%) (P = 0.045).	Gene
Bidari et al.	2022	Case-control	Iran	New diagnosis of FM according to 2016 ACR criteria	Non-FM chronic pain and no concurrent diagnosis of FM	73	50	46.7 ± 10.2	NR	100	Patients with FM tended to have lower serum BDNF levels compared to controls (5293.5 ± 2676.3 pg/ml vs. 6136.3 ± 4037.6; P = 0.77).	Serum
Cardinal et al.	2019	Cohort	Brazil	FM diagnosis according to 2016 ACR and VAS ≥50mm on most days of the last three months	Absence of any diagnosis of acute or chronic disease or medication use	45	17	46.3 ± 11.6	26.0 ± 5.0	100	BDNF levels were significantly higher in patients with FM compared to healthy controls (49.82 ± 16.31 ng/ml vs. 18.04 ± 10.19; P<0.001).	Serum
Caumo et al.	2016	Cross-sectional	Brazil	FM diagnosis according to 2010 ACR criteria	Pain-free controls without the use of medications	33	19	42.8 ± 9.7	28.1 ± 5.7	100	Higher levels of serum BDNF in patients with FM (50.78 ± 16.06 ng/ml) compared to healthy controls (19.00 ± 8.79).	Serum
Deitos et al.	2018	RCT	Brazil	FM diagnosis according to 2010 ACR criteria	Pain-free healthy controls	27	17	48.0 ± 9.0	28.7 ± 6.3	100	Serum BDNF levels were significantly higher in patients with FM compared to healthy controls (49.8 + 16.3 ng/ml vs. 14.8 ± 6.9; P<0.01).	Serum
Elkfury et al.	2021	Cross-sectional	Brazil	FM diagnosis according to 2010 ACR criteria and VAS ≥ 40mm associated with disability	Pain-free healthy controls without the use of medications	39	20	-	27.2 ± 4.7	100	Compared to healthy controls, patients with FM had higher serum BDNF levels (27.5 ± 4.1 ng/ml vs. 23.7 ± 6.2; P = 0.029).	Serum
Fawzy et al.	2015	Cross-sectional	Egypt	FM diagnosis according to 2010 ACR criteria	Age- and sex-matched healthy volunteers	50	30	34.0 ± 1.31	NR	86	Patients with FM had significantly higher levels of BDNF compared to healthy controls (25031.5 ± 3684.29 ng/ml vs. 13526.1 ± 3394.35).	Serum
Haas et al.	2010	Cross-sectional	Brazil	FM diagnosis according to 2006 ACR criteria	Age- and sex-matched healthy controls	60	30	46.3 ± 9.3	NR	100	Higher plasma BDNF levels in patients with FM compared to the control group (167.1 ± 171.2 pg/ml vs. 113.8 ± 149.6; P = 0.049).	Plasma
Iannuccelli et al.	2022	Cross-sectional	Italy	FM diagnosis according to 2016 ACR criteria	Sex-matched healthy controls	80	40	48.2 ± 8.8	25.1 ± 2.1	50	BDNF levels were significantly lower in FM patients compared to healthy controls (3.25 ± 1.96 ng/ml vs. 8.5 ± 3.6; P<0.001).	Serum
Jablochkova et al.	2019	RCT	Sweden	FM diagnosis according to 1990 ACR criteria	Age-matched and pain-free women.	100	75	50.0 ± 8.5	26.8 ± 4.7	100	Plasma levels of BDNF were significantly higher in patients with FM compared to healthy controls (1553.3 [2323.8] pg/ml vs. 671.6 [2250.2]; P = 0.001).	Plasma
Laske et al.	2007	Pilot	Germany	FM diagnosis according to 1990 ACR criteria	Controls without known major depression, depressive episode or use of antidepressant	86	41	51.2 ± 9.6	NR	90.7	Serum BDNF levels were significantly higher in patients with FM (19.6 ± 3.1 ng/ml vs. 16.8 ± 2.7; P<0.0001).	Serum
Martin et al.	2018	Case-control	Brazil	FM diagnosis according to 1990 ACR criteria	Chronic pain in the same age group of patients	24	13	45.9 ± 4.3	NR	100	In pre-treatment measurement, BDNF plasma concentrations had no significant difference (40.25 ± 16.35 ng/ml in controls and 46.57 ± 13.45 in patients with FM; P = 0.389)	Plasma
Nugraha et al.	2013	Cross-sectional	Germany	FM diagnosis according to 1990 ACR criteria	Age-matched healthy controls without chronic pain	55	28	53.7 ± 1.1	NR	100	BDNF concentration was significantly higher in patients with FM compared to healthy controls (15,557.6 [13218.20] pg/ml vs. 20,814.00 [7,152.75]; P <0.05).	Serum
Park et al.	2018	Cross-sectional	Korea	FM diagnosis according to 1990 ACR criteria	No history of chronic pain without matching for age or sex	827	408	46.8 ± 11.7	NR	93.6	No significant difference was seen in genotype and allele prevalence between patients with FM and healthy controls.	Gene
Polli et al.	2020	Cross-sectional	Belgium	FM diagnosis according to modified 2010 ACR criteria	Matched for age and physical activity healthy controls	54	28	49.9 ± 10.2	24.5 ± 4.1	100	BDNF protein expression was significantly higher in patients with FM (17.75 ± 4.48 ng/ml vs. 14.89 ± 3.55; P = 0.003). In addition, no significant between-groups differences were showed in the prevalence of gene polymorphism. No association between polymorphism and protein expression were showed.	Serum and Gene
Ranzolin et al.	2016	Cross-sectional	Brazil	FM diagnosis according to 2010 and 2016 ACR criteria	Age-matched healthy controls without acute or chronic pain	130	69	44.3 ± 6.6	NR	100	Study showed no difference between serum BDNF concentration of patients with FM and healthy controls (FM: 30.8 [21.8–37.1] vs. controls: 30.7 [21.1–35.4]; P = 0.595).	Serum
Sarchielli et al.^†^	2007	Cross-sectional	Italy	FM diagnosis according to 1990 ACR criteria	Age-matched controls not taking medications for at least two months	40	20	40.8 ± 5.6	NR	22.5	Significantly higher levels of CSF BDNF levels were found in FM patients compared to controls (39.4 ± 6.7 pg/ml vs. 11.3 ± 3.4; P<0.001)	CSF
Stefani et al.	2019	Cross-sectional	Brazil	FM diagnosis according to 2010 ACR and VAS ≥40mm that lasted more than three months with functional disability	Pain free volunteers without CNS complications	96	79	-	NR	100	Higher BDNF levels were seen in patients with FM compared to healthy controls (41.23 ± 20.03 pg/ml vs. 20.49 ± 8.82).	Serum
Taşkin et al.	2008	Cross-sectional	Turkey	Previously diagnosed FM	Healthy controls	45	19	38.1 ± 7.8	NR	86.7	Non-significant lower levels of BDNF were found in FM patients (30.75 ± 8.94 ng/ml) compared to healthy controls (31.42 ± 8.85).	Serum
Xiao et al.	2012	Cross-sectional	United states	FM diagnosis according to ACR criteria	Healthy controls that they had not sought medical care because of pain during the previous 5 years	153	95	47.3 ± 11.0	32.8 ± 9.1	89.5	The study found that genotype and allele frequency were not significantly different between patients with FM and healthy controls.	Gene

^†^ This study was not included in meta-analyses. FM, fibromyalgia; ACR, American College of Rheumatology; VAS, visual analog scale; BDNF, brain-derived neurotrophic factor; RCT, randomized controlled trial; CSF, cerebrospinal fluid; CNS, central nervous system.

**Table 2:** Quality Assessment of Included Studies Based on Newcastle-Ottawa Scale (NOS).

Study	Selection				Comparability	Outcome		Overall Score
	Representation	Sample size	Non-Respondents	Exposure		Outcome	Statistical test	
Alves et al. (2020)	*	*	*	**	**	**	*	10
Bidari et al. (2022)	*	*	*	**	-	**	*	8
Cardinal et al. (2019)	*	*	*	**	-	**	*	8
Caumo et al. (2016)	*	*	*	**	-	**	*	8
Deitos et al. (2018)	*	*	*	**	-	**	*	8
Elkfury et al. (2021)	*	*	*	**	**	**	*	10
Fawzy et al. (2015)	*	*	*	**	**	**	*	10
Haas et al. (2010)	*	*	*	**	**	**	*	10
Iannuccelli et al. (2022)	*	*	*	**	**	**	*	10
Jablochkova et al. (2019)	*	*	*	**	*	**	*	9
Laske et al. (2007)	*	*	*	**	**	**	*	10
Martin et al. (2018)	*	*	*	**	-	**	*	8
Nugraha et al. (2013)	*	*	*	**	*	**	*	9
Park et al. (2018)	*	*	*	**	-	**	*	8
Polli et al. (2020)	*	*	*	**	**	**	*	10
Ranzolin et al. (2016)	*	*	*	**	*	**	*	9
Sarchielli et al. (2007)	*	*	*	**	*	**	*	9
Stefani et al. (2019)	*	*	*	**	-	**	*	8
Taşkin et al. (2008)	*	*	*	**	-	**	*	8
Xiao et al. (2022)	*	*	*	**	-	**	*	8

### 3.2. Circulatory BDNF levels in patients with FM vs. controls

#### 3.2.1. Meta-analysis and subgroup analysis

**Fig 2:**
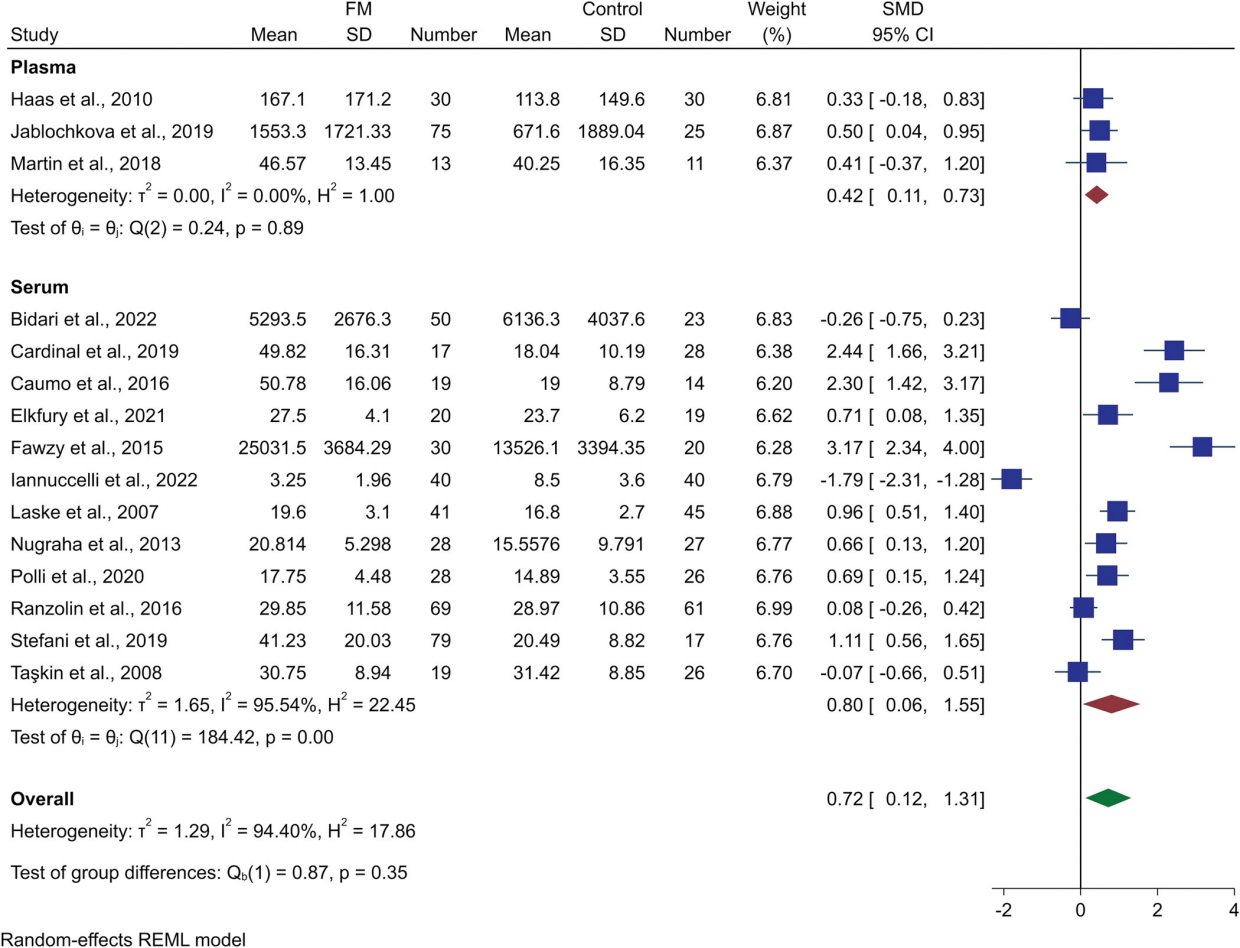
Forest plot representing the meta-analysis and subgroup analysis (serum/plasma) of BDNF levels in fibromyalgia patients compared to controls.

Fifteen studies assessing blood (serum or plasma) BDNF levels were used for the meta-analysis. The studies by Deitos et al. [43] and Cardinal et al. [40] had the same population of FM patients; therefore, we included the latter due to the larger sample size. The meta-analysis found that BDNF was significantly higher in FM compared to controls (SMD 0.72, 95% CI 0.12 to 1.31; p-value = 0.02; I^2^ = 94.40%; Fig 2). This result had a high level of heterogeneity.

Subgroup analysis based on serum or plasma BDNF levels was conducted, and three and 12 studies measured peripheral BDNF levels, respectively. Both plasma-measured and serum-measured BDNF were statistically higher in FM patients (SMD 0.42, 95% CI 0.11 to 0.73, I^2^ = 0%; and SMD 0.80, 95% CI 0.06 to 1.55, I^2^ = 95.54%, respectively) (Fig 2). As a result of this subgroup analysis, the heterogeneity (I^2^) was reduced in the plasma subgroup of studies.

**Fig 3:**
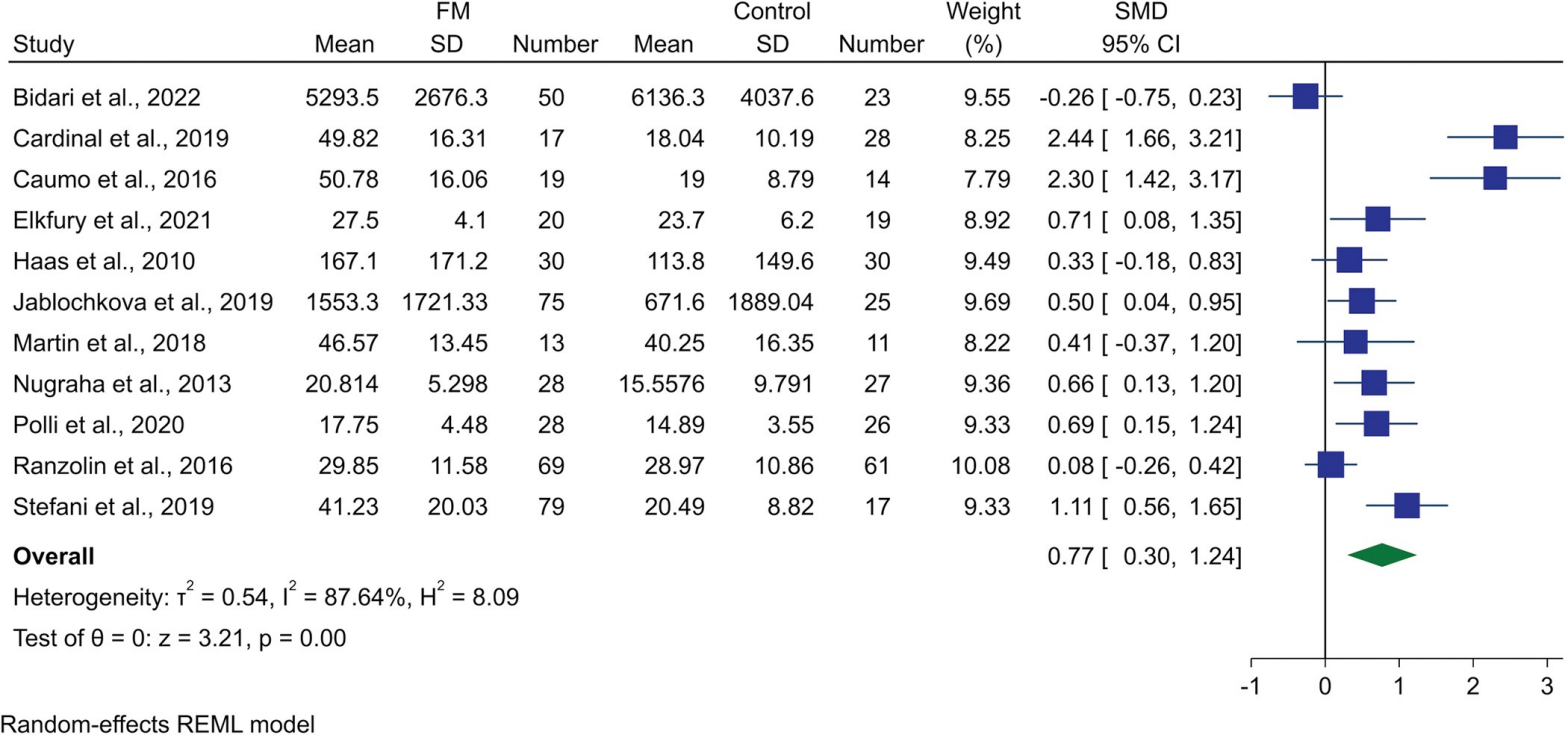
Forest plot representing the female subgroup meta-analysis of BDNF levels in fibromyalgia patients compared to controls.

A subgroup of studies that focused on BDNF levels of female patients was analyzed. From the 10 studies, despite high heterogeneity, it was shown that female FM patients had significantly higher peripheral BDNF compared to healthy female controls (SMD 0.77, 95% CI 0.30 to 1.24; p-value <0.01, I^2^ = 87.64%, Fig 3). Moderate quality of analyses was observed based on the GRADE approach, due to high inconsistency which stems from the high heterogeneity observed (Table 3).

**Table 3:** Summary of the GRADE quality of evidence assessment.

Quality Assessment						Number of Patients		SMD/OR (95% CI)	Quality
# Studies	Risk of Bias	Inconsistency	Indirectness	Imprecision	Publication Bias	FM	Control		
**FM vs. Healthy Controls (All studies)**									
15	Not serious	Serious	Not serious	Not serious	Not serious	558	412	SMD	⊕⊕⊕⊕ Moderate^†^
								0.72	
								[0.12, 1.31]	
**FM vs. Healthy Controls (Plasma)**									
3	Not serious	Not serious	Not serious	Not serious	Not serious	118	66	SMD	⊕⊕⊕⊕ Mild
								0.42	
								[0.11, 0.73]	
**FM vs. Healthy Controls (Serum)**									
12	Not serious	Serious	Not serious	Not serious	Not serious	440	346	SMD	⊕⊕⊕⊕ Moderate^†^
								0.80	
								[0.06, 1.55]	
**FM vs. Healthy Controls (Female)**									
11	Not serious	Serious	Not serious	Not serious	Not serious	428	281	SMD	⊕⊕⊕⊕ Moderate^†^
								0.77	
								[0.30, 1.24]	
**Gene Polymorphism**									
4	Not serious	Serious	Not serious	Not serious	Not serious	NA	NA	OR	⊕⊕⊕⊕ Moderate^†^
								1.31	
								[0.66, 2.58]	

**Abbreviations:** CI: confidence interval, SMD: Standardized Mean Difference, OR: Odds Ratio, NA: Not Applicable

^†^Moderate due to serious inconsistencies and high heterogeneity in meta-analysis

#### 3.2.2. Sensitivity analysis and outlier detection

Sensitivity leave-one-out analysis was conducted by omitting each of the individual studies for BDNF levels, without affecting the overall pooled result. Galbraith plot was designed for BDNF levels and gene polymorphism. For the former one, the studies by Fawzy et al. [51] and Iannuccelli et al. [28] had an outlier effect (S1 Fig). The analysis without this study revealed a significantly higher peripheral BDNF level in FM patients (SMD 0.72, 95% CI 0.31 to 1.13; p-value <0.01) (S2 Fig).

#### 3.2.3. Publication bias

The funnel plot did not show any apparent asymmetry for BDNF levels meta-analysis in patients with FM vs. healthy controls (S3 Fig). However, both Egger’s and Begg’s statistical tests suggest the presence of publication bias in BDNF-level analysis (p-value <0.01 and p-value = 0.04, respectively).

#### 3.2.4. Meta-regression

We conducted meta-regression analyses to explore the potential sources of the variance observed among the studies included in the analysis. The univariable meta-regression model did not determine any relationship between sample size, publication year, or age with either BDNF levels. In addition, the sample size and age variables accounted for 4.69% and 6.90% of the observed variance among the studies, respectively (S3 Table and S4–S6 Figs).

### 3.3. BDNF gene polymorphism in FM patients vs. controls

#### 3.3.1. Meta-analysis

**Fig 4:**
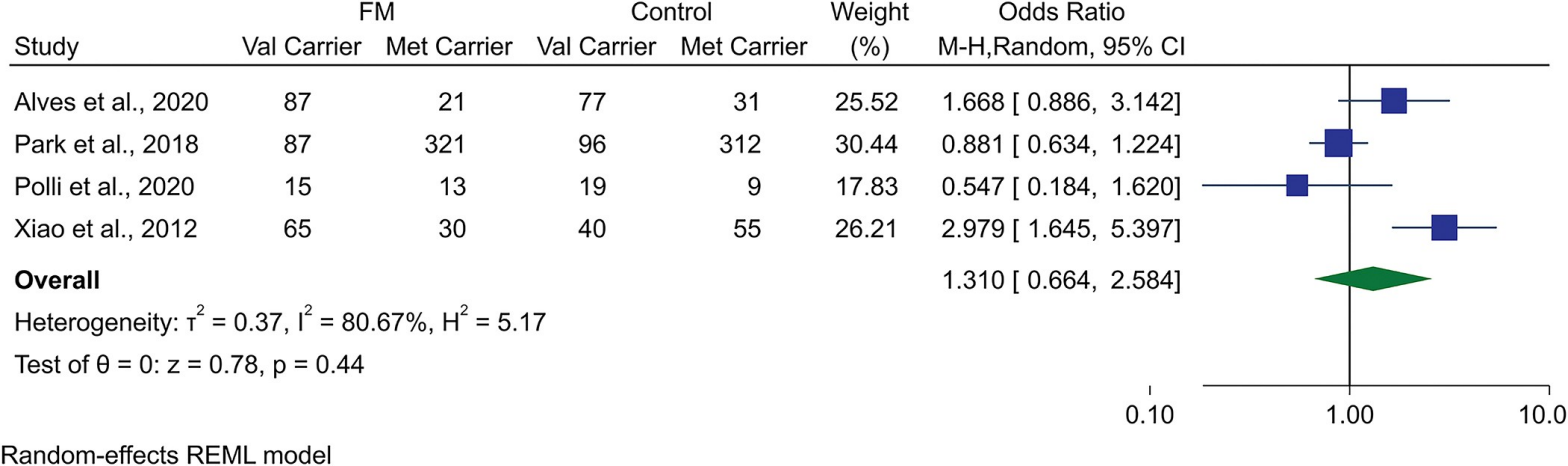
Forest plot representing the meta-analysis of BDNF gene Val66Met polymorphism in fibromyalgia patients compared to controls.

The Val/Met gene polymorphism at the rs6265 site was analyzed as the most gene polymorphism investigated in FM. There was no difference between the rate of Val/Met carrier status in FM patients and healthy controls in spite of higher Val carriers in FM patients (OR 1.31, 95% CI 0.67 to 2.58, p-value = 0.43, I^2^: 80.56%). This was associated with high heterogeneity (Fig 4). Table 3 shows the GRADE assessment of evidence quality which shows moderate quality for this analysis on gene polymorphisms.

#### 3.3.2. Sensitivity analysis and outlier detection

Sensitivity analysis by the leave-one-out method did not show any effect on the overall pooled estimate in BDNF gene polymorphism meta-analysis. Moreover, the Galbraith plot for the polymorphism did not show any outlier (S7 Fig).

#### 3.3.3. Publication bias

Funnel plots did not show any apparent asymmetry for this meta-analysis (S8 Fig). Similarly, no significant evidence of publication bias was observed for gene polymorphism analysis (p-value = 0.63 and p-value = 1.00, respectively).

#### 3.3.4. Meta-regression

In the gene polymorphism analysis, none of the variables, including sample sizes, mean age, or publication year had a significant relationship with the pooled effect size. Moreover, none of the variables could reduce the variance except for the publication year, which reduced the total variance by 38.29% (S3 Table and S9–S11 Figs).

### 3.4. CSF levels of BDNF in FM and controls

Only one study investigated the BDNF CSF levels in the FM [49]. In the comparison between 20 primary FM syndrome patients and 20 healthy controls, BDNF was higher in FM patients in comparison with controls (47.2 ± 2.65 pg/ml vs. 13.7 ± 2.7 pg/ml, p-value <0.001).

### 3.5. BDNF and population characteristics

None of the studies that evaluated the relationship between BDNF levels and age in patients with FM found any significant correlation [39, 46, 47, 50–52, 54, 56]. Iannuccelli and colleagues [28] reported that male FM patients had significantly lower levels of serum BDNF compared to female FM patients (4.72 ± 2.4 vs 1.82 ± 1.4, p-value < 0.0001). On the other hand, Laske et al. [47] and Taskin et al. [50] found no significant gender differences in FM patients (p-value = 0.8, p-value = 0.935).

Three studies conducted by Haas et al. [56], Laske et al. [47], and Nugraha et al. [52] revealed that there was no significant correlation between BDNF levels (in plasma, serum, and serum, respectively) and illness duration in patients with FM (r = -0.05; p-value = 0.79, r = 0.1; p-value = 0.3, r = 0.115; p-value = 0.498). However, another study by Sarchielli et al. [49] showed that CSF levels of BDNF were significantly correlated with the duration of FM (years) and the number of days with pain per month (r = 0.57, p-value = 0.01, and r = 0.55, p-value = 0.02).

### 3.6. BDNF and quality of life

Bidari et al. [39] found that as the serum level of BDNF decreased, the scores of several scales evaluating a patient’s quality of life increased, including the pain Visualized Analog Scale (VAS) and the Polysymptomatic Distress Scale (PSD). Haas et al. [56] discovered no significant relationship between plasma BDNF level and VAS (r = -0.12, p-value = 0.50), number of tender points (r = -0.02, p-value = 0.89), or Hamilton Depression (HAM-D) score (r = -0.14, p-value = 0.44). Likewise, Taskin et al. [50] found that serum BDNF level had no significant correlation with VAS (r = 0.191; p-value = 0.204) and HAM-D scores (r = 0.085; p p-value 0.579). Another study by Sarchielli and colleagues found no significant relationship between BDNF levels in CSF and VAS values, FIQ, brief pain inventory (short form) pain severity, pain threshold, and the number of tender points [49].

According to a study by Fawzy et al. [51], the mean serum BDNF level has a positive correlation with the grade of depression. On the contrary, Laske et al. [47] found no significant difference in mean BDNF serum concentrations between patients with and without recurrent major depression (p-value = 1.0). Polli et al. [46] performed a repeated-measure regression model showing that serum BDNF predicts chronic fatigue syndrome (CFS) symptoms list scores (F = 14.410, t = 3.796, 95% CI = 1.79 to 5.71, p-value = 0.001) and widespread hyperalgesia (F = 4.147, t = 2.036, 95% CI = 0.01 to 0.08, p-value = 0.044). Another study conducted by Nugraha et al. [52] reported that serum BDNF level in FM patients significantly increases with depression level according to the Hospital Anxiety and Depression Scale (HADS). Despite that, serum levels of BDNF were not significantly correlated with anxiety scores in HADS.

### 3.7. BDNF and medical therapies

Bidari and colleagues reported that a one-month treatment with duloxetine significantly reduced serum BDNF levels, even after adjusting for depression, pain, and severity of the disease in their linear mixed model (p < 0.01) [39]. Cardinal et al. found that serum BDNF has a positive correlation with analgesics use (R^2^ = 0.54, β coefficient = 20.94, CI 95% = 9.84 to 32.04) and with anticonvulsant use (R^2^ = 0.54, β coefficient = 22.71, CI 95% = 8.19 to 37.22), and a negative correlation with selective serotonin reuptake inhibitors (SSRIs) use (R^2^ = 0.38, β coefficient = −14.50, 95% CI −26.43 to −2.56) [40]. In contrast, Deitos et al. [43] found a negative correlation between serum BDNF and weekly analgesic use (R^2^ = 0.24, β coefficient = −0.42, p-value < 0.01). Haas et al. [56] found that there were no significant differences in BDNF levels between patients taking no antidepressant treatments, patients taking analgesic doses of antidepressants, and patients taking antidepressants at therapeutic doses for depression (in pg/mL: group 1 = 130.4 ± 106.2; group 2 = 146.6 ± 137.9; group 3 = 194.0 ± 210.3, p-value = 0.81).

A trial conducted by Jablochkova and colleagues found no significant changes in BDNF levels in FM patients, before and after undergoing resistance exercise [55]. However, VAS global pain, Pain Catastrophizing Scale (PSC), Fibromyalgia Impact Questionnaire (FIQ), and Multidimensional Fatigue Inventory (MFI) scores changed significantly during this intervention.

Martin et al. [54] conducted a study evaluating the effect of aquatic physical therapy on BDNF levels in women with FM. They found that BDNF pre-intervention values showed a significant reduction from session 1 to session 10 in patients with FM (p-value = 0.002).

### 3.8. BDNF and motor cortex inhibition

In the study conducted by Cardinal and colleagues, BDNF adjusted index in patients with FM (adjusted with the use of SSRIs, anticonvulsants, and analgesics) was found to not correlate (analysis power = 96%) with short intracortical inhibition (SICI), intracortical facilitation (ICF), and the change on the numerical pain scale (NPS) during the conditioned pain modulation test (CPM-test) [40]. Deitos and colleagues performed a multiple-regression analysis showing that the cortical silent period (CSP) after using pregabalin is positively associated with the BDNF level before pregabalin [43]. However, the authors did not observe an interaction between using pregabalin and the BDNF level.

## 4. Discussion

In this study, we demonstrated that BDNF level was significantly higher in FM patients than in the non-FM control group, however, no significant difference was detected between the rate of Val/Met carrier status at the rs6265 site in FM patients and healthy controls. Furthermore, the results of the included studies regarding the association of BDNF level with sex, quality of life, illness duration, depression, and the use of various medications (e.g., analgesics, antidepressants, etc.) in patients with FM were controversial. Our study’s findings could help researchers in exploring the role of BDNF in FM and its possible clinical use.

Laske et al. conducted the first study on 41 FM patients and 45 healthy controls to evaluate the association of BDNF level with FM. Their result showed an increased BDNF in FM patients in comparison with healthy controls, which was independent of age, gender, and illness duration [47]. The serum BDNF has been investigated in several clinical manifestations of FM and also seems to be associated with some, such as depression, mood disorders, and dysfunctional eating behavior [41, 61–63].

As mentioned before, the pathophysiology of FM is still unknown; nevertheless, many mechanisms involved in FM pathophysiology have been suggested. The primary changes seen in FM are dysfunctions in monoaminergic neurotransmission, which result in increased excitatory neurotransmitter levels, including substance P and glutamate, and reduced norepinephrine and serotonin levels at the descending anti-nociceptive pathways of the spinal cord [64]. BDNF is one of the stimulatory factors for glutamate release [65, 66] and strengthens glutamatergic synapses [23], which could explain its role in FM. Furthermore, central sensitization is considered maladaptive neuroplasticity and one of the mechanisms implicated in the pathogenesis of FM [67]. BDNF regulates neuroplasticity and is involved in the sensitization of pain pathways at different levels, including the dorsal root ganglia, peripheral nociceptors, spinal dorsal horn neurons, and brain descending inhibitory and facilitatory pathways [16]. BDNF induces durable dorsal horn excitability, an underlying mechanism in the central sensitization [68]. Thus, BDNF could promote FM by contributing to central sensitization.

Therefore, patients with FM could benefit from the drugs targeting different neurotransmitter systems and BDNF levels. Bidari et al. showed that duloxetine–a serotonin and norepinephrine recapture inhibitor known to modulate BDNF levels [69]–improved the results of several FM and pain questionnaires in these patients; FM patients had decreased levels of BDNF after duloxetine treatment [39]. In FM patients who received thermal therapy (mud-bath or balneotherapy), pain reduction and improvement in the results of FIRQ and SF36 occurred; thermal therapy reduced BDNF concentration but did not change oxytocin levels, adenosine triphosphate (ATP) levels, and serotonin transporter in patients with FM [70]. Gentle touch therapy also caused pain reduction in patients with FM, which resulted from decreased BDNF levels [71]. These results go in line with reports of decreased BDNF levels with effective antipain treatments in different clinical contexts [72].

Since family history is a risk factor for FM, different studies have evaluated the role of genetic and gene polymorphism in this disease. In a study by Park et al. on BDNF gene polymorphism, the GG genotype (rs11030104) was represented to be a protective factor against this syndrome [45]. In our study, all reports but one [44] found that *BDNF* Val66Met (rs6265) polymorphism does not have an association with FM.

We considered both sources of BDNF in the circulatory system (plasma and serum) in our meta-analysis as they are highly correlated. We also ran subgroup analyses considering different biological matrices (i.e., serum vs. plasma). Average serum BDNF levels are greater than plasma levels [73, 74]. This difference results from the platelets’ degranulation at the time of the clotting process since reasonable amounts of BDNF have been identified in human platelets [75, 76]. In contrast to plasma levels, serum BDNF levels mostly depend on the clotting time, which varies among studies [77]. Despite these differences, the results were similar in serum- and plasma-measured BDNF levels.

While being the first study to investigate BDNF in FM, both in peripheral levels and gene polymorphisms, our study had several limitations. Significant heterogeneity across the studies perhaps due to different regimens, center settings, and populations might be a limiting factor that could affect the generalizability of our findings. We tried to minimize this heterogeneity by performing subgroup analyses and meta-regression, for which some factors could reduce observed heterogeneity such as the plasma subgroup or publication year meta-regression. Additionally, the inherent limitations of included studies which were inevitable should be taken into consideration. Inappropriate matching for confounding baseline features such as comorbidities in the included studies may have affected the results. The limited number of studies in gene polymorphism analysis and subgroup analyses can increase the overall risk of bias. Also, we excluded studies that did not mention BDNF concentrations and only reported it graphically [78, 79]. Moreover, different kits used in different studies might introduce bias into our analysis since some kits measure proBDNF in addition to BDNF [80], despite not mentioning it in detail. It should be emphasized that proBDNF levels might differ among different ethnicities, highlighting the need for using new, commercial human BDNF ELISA kits [63]. FM diagnosis according to different ACR criteria published in different years was another limitation. In order to include the studies that reported median and interquartile ranges, we had to convert them to mean with SDs [33, 34], which could be a source of bias in our results. As the results of different studies on the association of disease-related factors, such as quality of life, the use of medications, depression, etc., with BDNF levels in FM patients were controversial, further research with larger sample sizes and similar outcome measures could allow combining the results and clarify the relationship between BDNF levels and the aforementioned outcomes. Finally, as visual inspection of the funnel plot for assessment of the publication bias is suggested to need at least 10 studies [35], publication bias for our meta-analysis of gene polymorphism might be limited, despite the fact that Begg’s and Egger’s tests did not show any publication bias.

## 5. Conclusion

In conclusion, the pooled meta-analysis of the available studies demonstrated high peripheral levels of BDNF in FM, while BDNF gene polymorphism was not associated with FM. This finding can support clinicians and researchers to further investigate the emerging role of BDNF in pain modulation, specifically in the FM context, and evaluate its potential as a diagnostic and prognostic biomarker. For that, longitudinal studies with larger and more representative samples of patients with FM, controlling for confounding factors (e.g., medical and psychiatric comorbidities) are needed.

## Supporting information

S1 Fig Galbraith plot for meta-analysis of BDNF levels in FM patients compared to controls. (PNG)

S2 Fig Forest for meta-analysis of BDNF levels in FM patients compared to controls after removing outliers. (PNG)

S3 Fig Funnel plot for meta-analysis of BDNF levels in FM patients compared to controls. (PNG)

S4 Fig Bubble plot for meta-regression of BDNF levels in FM patients compared to controls based on the sample size. (PNG)

S5 Fig Bubble plot for meta-regression of BDNF levels in FM patients compared to controls based on mean age of participants. (PNG)

S6 Fig Bubble plot for meta-regression of BDNF levels in FM patients compared to controls based on publication year. (PNG)

S7 Fig Galbraith plot for meta-analysis of BDNF gene polymorphism in FM patients compared to controls. (PNG)

S8 Fig Funnel plot for meta-analysis of BDNF gene polymorphism in FM patients compared to controls. (PNG)

S9 Fig Bubble plot for meta-regression of BDNF gene polymorphism in FM patients compared to controls based on the sample size. (PNG)

S10 Fig Bubble plot for meta-regression of BDNF gene polymorphism in FM patients compared to controls based on the mean age of participants. (PNG)

S11 Fig Bubble plot for meta-regression of BDNF gene polymorphism in FM patients compared to controls based on publication year. (PNG)

S1 Table PRISMA 2020 checklist for performing systematic review. (DOCX)

S2 Table The search queries used for each database and the search results. (DOCX)

S3 Table Meta-regression of BDNF levels/gene polymorphism in FM patients and healthy controls. (DOCX)
