# Supplementary figures and images for: Brain-derived neurotrophic factor in fibromyalgia: A systematic review and meta-analysis of its role as a potential biomarker
Source: PLoS One. 2023 Dec 21;18(12):e0296103. doi: 10.1371/journal.pone.0296103 (PMC10734974; doi:10.1371/journal.pone.0296103)

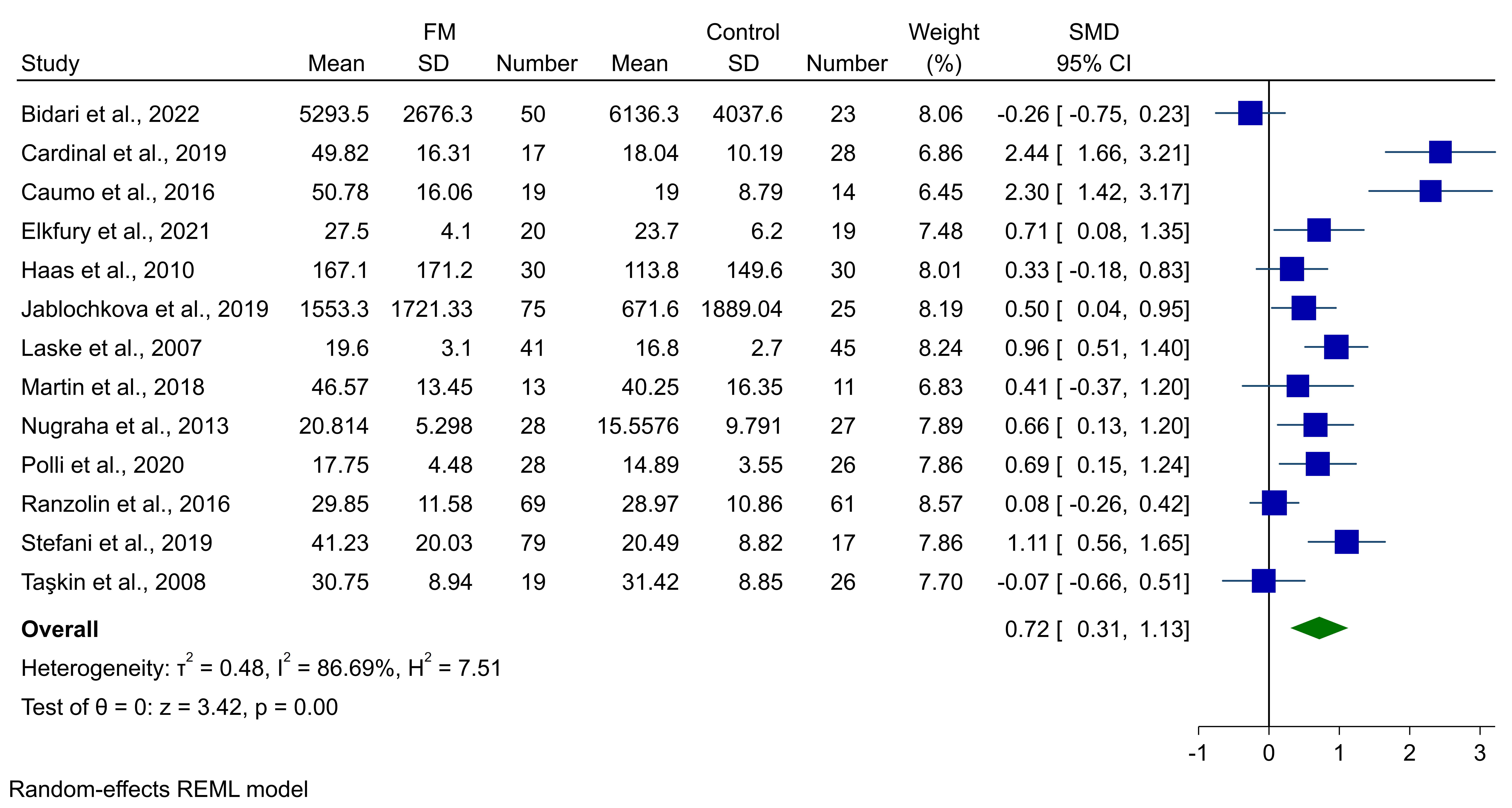

Supplement: S2 Fig — (PNG) [file pone.0296103.s002.png]

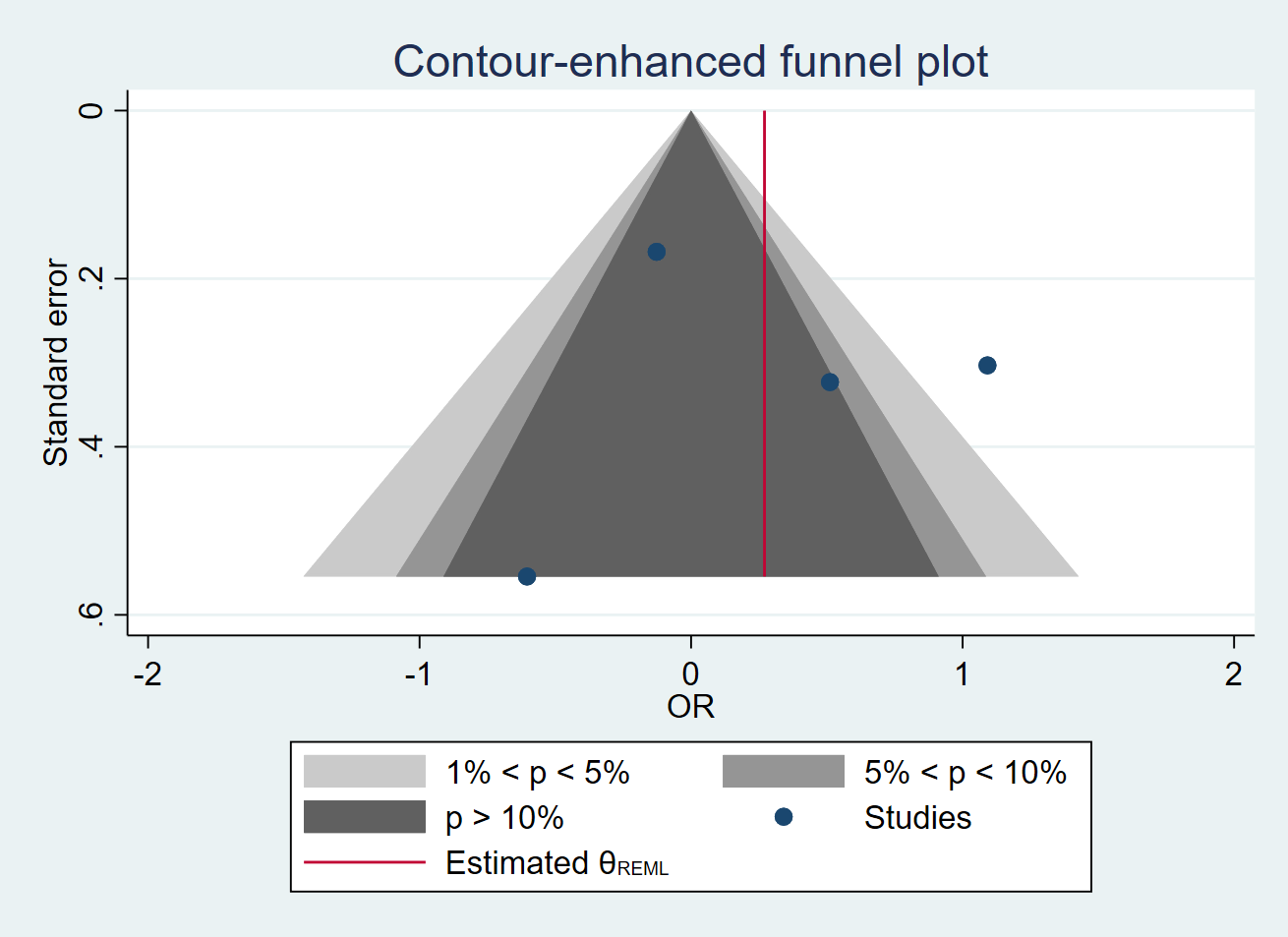

Supplement: S8 Fig — (PNG) [file pone.0296103.s008.png]

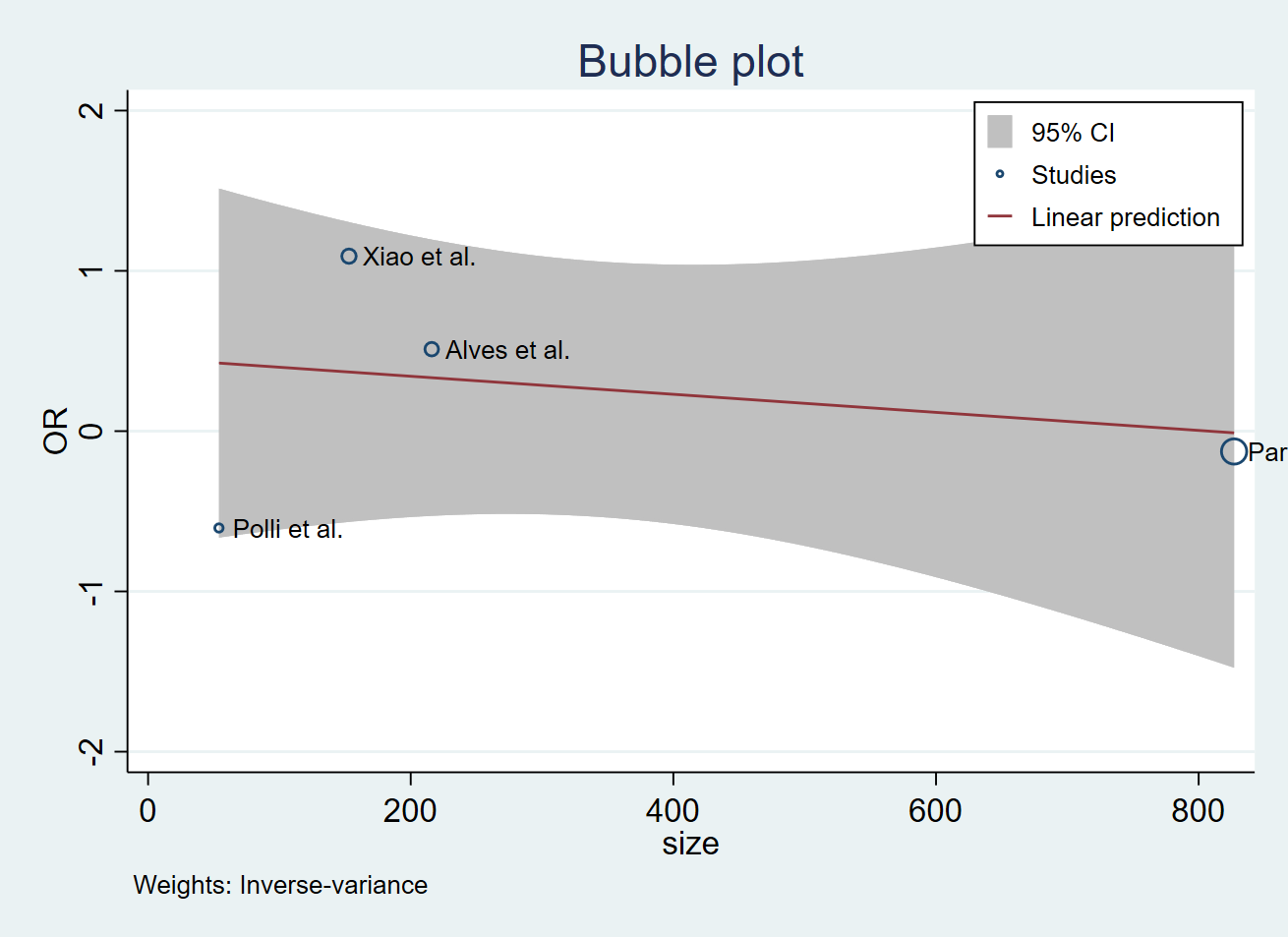

Supplement: S9 Fig — (PNG) [file pone.0296103.s009.png]

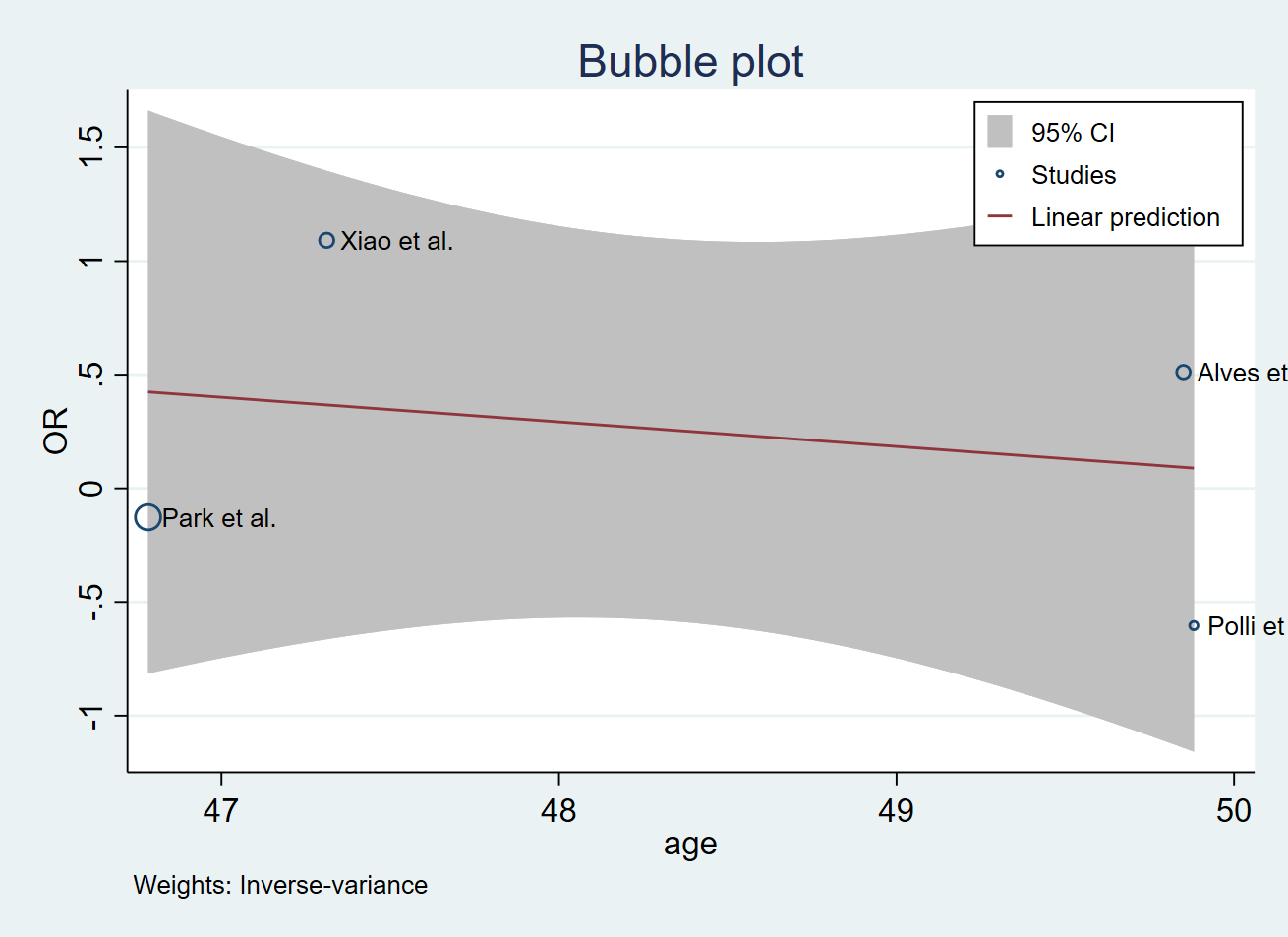

Supplement: S10 Fig — (PNG) [file pone.0296103.s010.png]

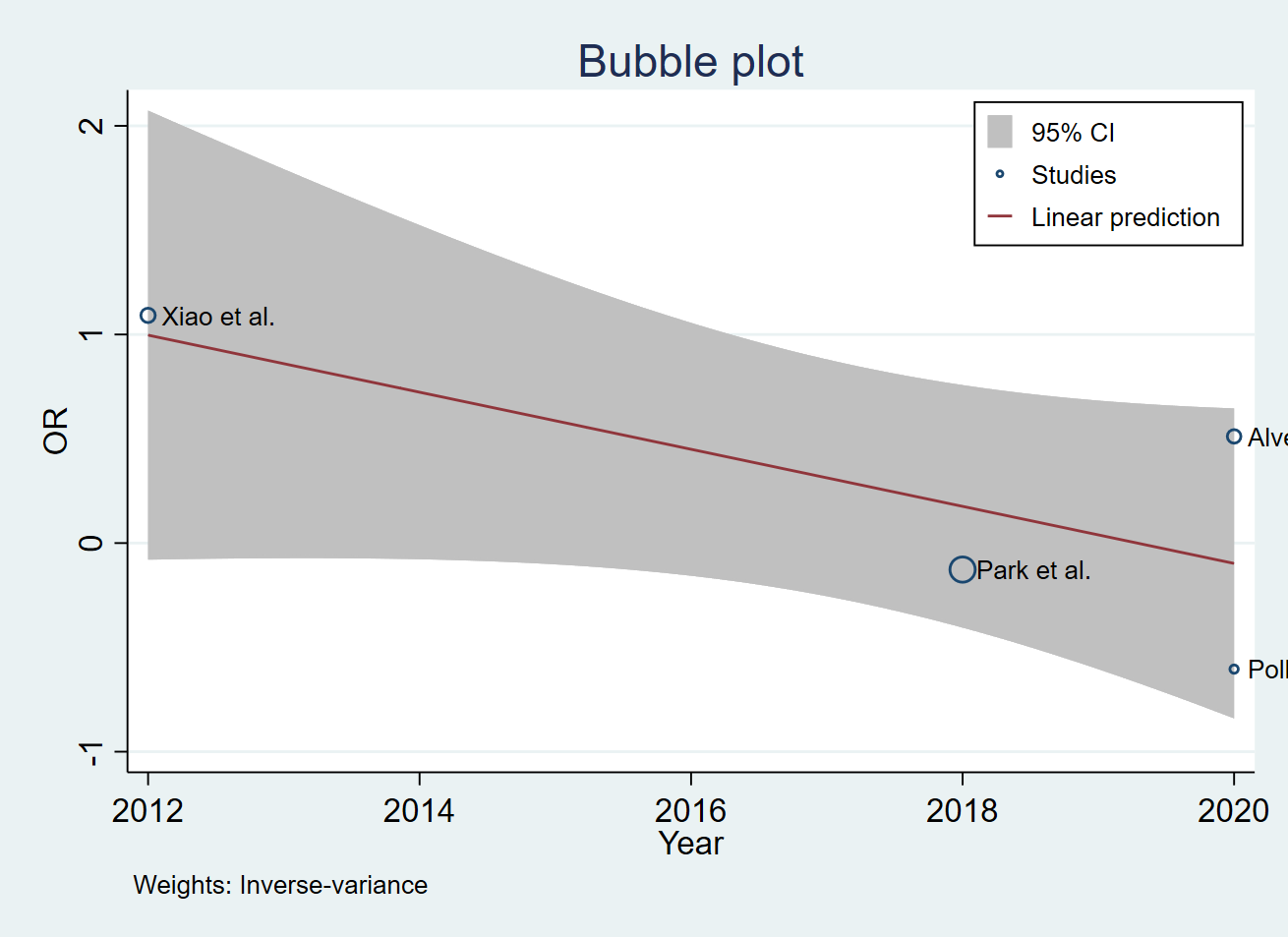

Supplement: S11 Fig — (PNG) [file pone.0296103.s011.png]
