# Supplementary material for: Brain-derived neurotrophic factor in fibromyalgia: A systematic review and meta-analysis of its role as a potential biomarker
Source: PLoS One. 2023 Dec 21;18(12):e0296103. doi: 10.1371/journal.pone.0296103 (PMC10734974; doi:10.1371/journal.pone.0296103)
Supplement: S2 Table — (DOCX) [file pone.0296103.s013.docx]

**S2 Table.** The search queries used for each database and the search results

|  | **Query** | **Results (No.)**  **July 4, 2023** |
| --- | --- | --- |
| **PubMed** | | |
| #1 | ("Brain-Derived Neurotrophic Factor"[Mesh] OR "BDNF"[tiab] OR "brain-derived neurotrophic factor"[tiab] OR "brain-derived neurotrophic factor"[tiab]) | 31,506 |
| #2 | (“fibromyalgia”[Mesh] OR “fibromyalgia*”[tiab] OR “Fibromyalgias”[tiab] OR “Fibromyalgia-Fibromyositis Syndrome”[tiab] OR “Fibromyalgia Fibromyositis Syndrome”[tiab] OR “Fibromyalgia-Fibromyositis Syndromes”[tiab] OR “Syndrome, Fibromyalgia-Fibromyositis”[tiab] OR “Syndromes, Fibromyalgia-Fibromyositis”[tiab] OR “Rheumatism, Muscular”[tiab] OR “Muscular Rheumatism”[tiab] OR “Fibrositis”[tiab] OR “Fibrositides”[tiab] OR “Myofascial Pain Syndrome, Diffuse”[tiab] OR “Diffuse Myofascial Pain Syndrome”[tiab] OR “Fibromyositis-Fibromyalgia Syndrome”[tiab] OR “Fibromyositis Fibromyalgia Syndrome”[tiab] OR “Fibromyositis-Fibromyalgia Syndromes”[tiab] OR “Syndrome, Fibromyositis-Fibromyalgia”[tiab] OR “Syndromes, Fibromyositis-Fibromyalgia”[tiab] OR “Fibromyalgia, Secondary”[tiab] OR “Fibromyalgias, Secondary”[tiab] OR “Secondary Fibromyalgia”[tiab] OR “Secondary Fibromyalgias”[tiab] OR “Fibromyalgia, Primary”[tiab] OR “Fibromyalgias, Primary”[tiab] OR “Primary Fibromyalgia”[tiab] OR “Primary Fibromyalgias”[tiab]) | 14,171 |
| #3 | #1 AND #2 | 53 |
| **Embase** | | |
| #1 | 'brain derived neurotrophic factor'/exp OR 'bdnf' OR 'brain-derived neurotrophic factor' | 54,610 |
| #2 | 'fibromyalgia'/exp OR 'fibromyalgia':ti,ab,kw OR 'fibromyalgia*':ti,ab,kw OR 'fibromyalgias':ti,ab,kw OR 'fibromyalgia-fibromyositis syndrome':ti,ab,kw OR 'fibromyalgia fibromyositis syndrome':ti,ab,kw OR 'fibromyalgia-fibromyositis syndromes':ti,ab,kw OR 'syndrome, fibromyalgia-fibromyositis':ti,ab,kw OR 'syndromes, fibromyalgia-fibromyositis':ti,ab,kw OR 'rheumatism, muscular':ti,ab,kw OR 'muscular rheumatism':ti,ab,kw OR 'fibrositis':ti,ab,kw OR 'fibrositides':ti,ab,kw OR 'myofascial pain syndrome, diffuse':ti,ab,kw OR 'diffuse myofascial pain syndrome':ti,ab,kw OR 'fibromyositis-fibromyalgia syndrome':ti,ab,kw OR 'fibromyositis fibromyalgia syndrome':ti,ab,kw OR 'fibromyositis-fibromyalgia syndromes':ti,ab,kw OR 'syndrome, fibromyositis-fibromyalgia':ti,ab,kw OR 'syndromes, fibromyositis-fibromyalgia':ti,ab,kw OR 'fibromyalgia, secondary':ti,ab,kw OR 'fibromyalgias, secondary':ti,ab,kw OR 'secondary fibromyalgia':ti,ab,kw OR 'secondary fibromyalgias':ti,ab,kw OR 'fibromyalgia, primary':ti,ab,kw OR 'fibromyalgias, primary':ti,ab,kw OR 'primary fibromyalgia':ti,ab,kw OR 'primary fibromyalgias':ti,ab,kw | 27,451 |
| #3 | #1 AND #2 | 137 |
| **Web of Science** | | |
| #1 | (TS="BDNF" OR TS="brain-derived neurotrophic factor" OR TS="brain-derived neurotrophic factor") | 36,362 |
| #2 | (TS=“fibromyalgia” OR TS=“fibromyalgia*” OR TS=“Fibromyalgias” OR TS=“Fibromyalgia-Fibromyositis Syndrome” OR TS=“Fibromyalgia Fibromyositis Syndrome” OR TS=“Fibromyalgia-Fibromyositis Syndromes” OR TS=“Syndrome, Fibromyalgia-Fibromyositis” OR TS=“Syndromes, Fibromyalgia-Fibromyositis” OR TS=“Rheumatism, Muscular” OR TS=“Muscular Rheumatism” OR TS=“Fibrositis” OR TS=“Fibrositides” OR TS=“Myofascial Pain Syndrome, Diffuse” OR TS=“Diffuse Myofascial Pain Syndrome” OR TS=“Fibromyositis-Fibromyalgia Syndrome” OR TS=“Fibromyositis Fibromyalgia Syndrome” OR TS=“Fibromyositis-Fibromyalgia Syndromes” OR TS=“Syndrome, Fibromyositis-Fibromyalgia” OR TS=“Syndromes, Fibromyositis-Fibromyalgia” OR TS=“Fibromyalgia, Secondary” OR TS=“Fibromyalgias, Secondary” OR TS=“Secondary Fibromyalgia” OR TS=“Secondary Fibromyalgias” OR TS=“Fibromyalgia, Primary” OR TS=“Fibromyalgias, Primary” OR TS=“Primary Fibromyalgia” OR TS=“Primary Fibromyalgias”) | 20,381 |
| #3 | #1 AND #2 | 74 |
| **SCOPUS** | | |
| #1 | (TITLE-ABS-KEY("Brain-Derived Neurotrophic Factor") OR TITLE-ABS-KEY("BDNF") OR TITLE-ABS-KEY("brain-derived neurotrophic factor") OR TITLE-ABS-KEY("brain-derived neurotrophic factor")) | 47,908 |
| #2 | (TITLE-ABS-KEY(“fibromyalgia”) OR TITLE-ABS-KEY(“fibromyalgia*”)OR TITLE-ABS-KEY(“Fibromyalgias”) OR TITLE-ABS-KEY(“Fibromyalgia-Fibromyositis Syndrome”) OR TITLE-ABS-KEY(“Fibromyalgia Fibromyositis Syndrome”) OR TITLE-ABS-KEY(“Fibromyalgia-Fibromyositis Syndromes”) OR TITLE-ABS-KEY(“Syndrome, Fibromyalgia-Fibromyositis”) OR TITLE-ABS-KEY(“Syndromes, Fibromyalgia-Fibromyositis”) OR TITLE-ABS-KEY(“Rheumatism, Muscular”) OR TITLE-ABS-KEY(“Muscular Rheumatism”) OR TITLE-ABS-KEY(“Fibrositis”) OR TITLE-ABS-KEY(“Fibrositides”) OR TITLE-ABS-KEY(“Myofascial Pain Syndrome, Diffuse”) OR TITLE-ABS-KEY(“Diffuse Myofascial Pain Syndrome”) OR TITLE-ABS-KEY(“Fibromyositis-Fibromyalgia Syndrome”) OR TITLE-ABS-KEY( “Fibromyositis Fibromyalgia Syndrome”) OR TITLE-ABS-KEY(“Fibromyositis-Fibromyalgia Syndromes”) OR TITLE-ABS-KEY(“Syndrome, Fibromyositis-Fibromyalgia”) OR TITLE-ABS-KEY(“Syndromes, Fibromyositis-Fibromyalgia”) OR TITLE-ABS-KEY(“Fibromyalgia, Secondary”) OR TITLE-ABS-KEY(“Fibromyalgias, Secondary”) OR TITLE-ABS-KEY(“Secondary Fibromyalgia”) OR TITLE-ABS-KEY(“Secondary Fibromyalgias”) OR TITLE-ABS-KEY(“Fibromyalgia, Primary”) OR TITLE-ABS-KEY(“Fibromyalgias, Primary”) OR TITLE-ABS-KEY(“Primary Fibromyalgia”) OR TITLE-ABS-KEY(“Primary Fibromyalgias”)) | 23,018 |
| #3 | #1 AND #2 | 113 |
| **Cochrane Library** | | |
| #1 | "BDNF" OR "brain-derived neurotrophic factor" | 2,191 |
| #2 | (“fibromyalgia” OR “fibromyalgia*” OR “Fibromyalgias” OR “Fibromyalgia-Fibromyositis Syndrome” OR “Fibromyalgia Fibromyositis Syndrome” OR “Fibromyalgia-Fibromyositis Syndromes” OR “Syndrome, Fibromyalgia-Fibromyositis” OR “Syndromes, Fibromyalgia-Fibromyositis” OR “Rheumatism, Muscular” OR “Muscular Rheumatism” OR “Fibrositis” OR “Fibrositides” OR “Myofascial Pain Syndrome, Diffuse” OR “Diffuse Myofascial Pain Syndrome” OR “Fibromyositis-Fibromyalgia Syndrome” OR “Fibromyositis Fibromyalgia Syndrome” OR “Fibromyositis-Fibromyalgia Syndromes” OR “Syndrome, Fibromyositis-Fibromyalgia” OR “Syndromes, Fibromyositis-Fibromyalgia” OR “Fibromyalgia, Secondary” OR “Fibromyalgias, Secondary” OR “Secondary Fibromyalgia” OR “Secondary Fibromyalgias” OR “Fibromyalgia, Primary” OR “Fibromyalgias, Primary” OR “Primary Fibromyalgia” OR “Primary Fibromyalgias”) | 4,606 |
| #3 | #1 AND #2 | 27 |
| **Total** | | 404 |
| **Total without duplicates** | | 200 |
