# Supplementary material for: Brain-derived neurotrophic factor in fibromyalgia: A systematic review and meta-analysis of its role as a potential biomarker
Source: PLoS One. 2023 Dec 21;18(12):e0296103. doi: 10.1371/journal.pone.0296103 (PMC10734974; doi:10.1371/journal.pone.0296103)
Supplement: S3 Table — (DOCX) [file pone.0296103.s014.docx]

**S3 Table**. Meta-regression of BDNF levels/gene polymorphism in FM patients and healthy controls

| **Moderator** | **No. of subjects** | | **Meta-regression** | | | | **R^2^ Analog (proportion of variance explained)** |
| --- | --- | --- | --- | --- | --- | --- | --- |
|  | **Fibromyalgia** | **Healthy control** | **Slope** | **95% Confidence interval** | | ***p-value*** |  |
| **BDNF concentration** | | | | | | | |
| **Sample size** | 558 | 412 | -0.0138 | -0.0344 | 0.0069 | 0.191 | 4.69% |
| **Age (mean, years)** | 558 | 412 | -0.0898 | -0.2096 | 0.0301 | 0.142 | 6.90% |
| **Publication year** | 558 | 412 | -0.0363 | -0.1649 | 0.0924 | 0.581 | 0% |
| **BDNF gene polymorphism (Valine/Methionine genotype)** | | | | | | | |
| **Sample size** | 639 | 611 | -0.0005 | -0.0031 | 0.0020 | 0.665 | 0% |
| **Age (mean, years)** | 639 | 611 | -0.1080 | -0.6991 | 0.4830 | 0.720 | 0% |
| **Publication year** | 639 | 611 | -0.1368 | -0.3114 | 0.0378 | 0.125 | 38.29% |
